# The Highly Repetitive Region of the *Helicobacter pylori* CagY Protein Comprises Tandem Arrays of an α-Helical Repeat Module

**DOI:** 10.1016/j.jmb.2008.01.053

**Published:** 2008-03-28

**Authors:** Robin M. Delahay, Graham D. Balkwill, Karen A. Bunting, Wayne Edwards, John C. Atherton, Mark S. Searle

**Affiliations:** 1 Institute of Infection, Immunity and Inflammation, Centre for Biomolecular Sciences, University of Nottingham, Nottingham NG7 2RD, UK; 2 Wolfson Digestive Diseases Centre, University of Nottingham, C Floor, South Block, Queen's Medical Centre, Nottingham NG7 2UH, UK; 3 School of Chemistry, Centre for Biomolecular Sciences, University of Nottingham, Nottingham NG7 2RD, UK; 4 Institute of Genetics, Queen's Medical Centre, University of Nottingham, Nottingham NG7 2UH, UK

**Keywords:** T4SS, type IV secretion system, PAI, pathogenicity island, TPR, tetratricopeptide repeat, PBS, phosphate-buffered saline, *Helicobacter pylori*, CagY, tetratricopeptide repeat, α-helical repeat, type IV secretion

## Abstract

The *cag*-pathogenicity-island-encoded type IV secretion system of *Helicobacter pylori* functions to translocate the effector protein CagA directly through the plasma membrane of gastric epithelial cells. Similar to other secretion systems, the Cag type IV secretion system elaborates a surface filament structure, which is unusually sheathed by the large *cag*-pathogenicity-island-encoded protein CagY. CagY is distinguished by unusual amino acid composition and extensive repetitive sequence organised into two defined repeat regions. The second and major repeat region (CagY_rpt2_) has a regular disposition of six repetitive motifs, which are subject to deletion and duplication, facilitating the generation of CagY size and phenotypic variants. In this study, we show CagY_rpt2_ to comprise two highly thermostable and acid-stable α-helical structural motifs, the most abundant of which (motif A) occurs in tandem arrays of one to six repeats terminally flanked by single copies of the second repeat (motif B). Isolated motifs demonstrate hetero- and homomeric interactions, suggesting a propensity for uniform assembly of discrete structural subunit motifs within the larger CagY_rpt2_ structure. Consistent with this, CagY proteins comprising substantially different repeat 2 motif organisations demonstrate equivalent CagA translocation competence, illustrating a remarkable structural and functional tolerance for precise deletion and duplication of motif subunits. We provide the first insight into the structural basis for CagY_rpt2_ assembly that accommodates both the variable motif sequence composition and the extensive contraction/expansion of repeat modules within the CagY_rpt2_ region.

## Introduction

*Helicobacter pylori* is a highly successful human pathogen that colonises the gastric mucosa of approximately 50% of the world's population. All infected individuals develop chronic gastritis, which, by itself, is asymptomatic. However, a subpopulation of infected human hosts develop a range of severe gastroduodenal diseases including duodenal ulceration and gastric cancer.^1,2^ Epidemiological studies indicate that these more severe clinical outcomes correlate with infection by *H. pylori* strains possessing a 40-kb pathogenicity island (PAI) termed *cag*.^3^ The *cag* PAI encodes the structural components of a putative type IV secretion system (T4SS), which functions to translocate the *cag*-PAI-encoded effector protein CagA into gastric epithelial cells.^3,4^ CagA has myriad profound effects on host cell signalling, severely disrupting both cell function and morphology as a consequence of phosphorylation-dependent and -independent interactions with multiple host proteins.^5–7^

Unlike the CagA protein, the secretion system that mediates its delivery to the gastric epithelium has been poorly studied. Amongst the 27–31 *cag*-encoded proteins are putative homologues of six core Vir proteins of the archetypal T4SS/T-DNA transfer system of *Agrobacterium tumefaciens*.^3,4^ A subset of these proteins including CagX/HP0528, CagT/HP0532, and CagY/HP0527 are reported to comprise a large filamentous extension to the T4SS elaborated on the surface of *H. pylori*,^8,9^ which differs from the smaller pili associated with other type IV systems. Although these proteins have discrete sequence similarity to Vir counterparts (CagX/VirB9, CagT/VirB7, and CagY/VirB10), their localisation to the extracellular filament structure, rather than integral to the membrane-spanning T4SS channel, appears divergent from the *A. tumefaciens* T4SS assembly.^10^

The Cag filament comprising at least CagX and CagT is irregularly sheathed by a processed form of the CagY protein.^8,9^ Filament elaboration and surface covering by CagY are indicated as components of host cell contact, since in the absence of host cells, *H. pylori* display reduced numbers of incompletely sheathed filaments.^9^ Isogenic *H. pylori* mutants deficient for *cagX*, *cagT*, and *cagY* have been shown to be abrogated in their ability to translocate CagA,^8,11^ and the ability of *cagX* and *cagY* mutants but not *cagA* mutants to colonise mice is severely impaired.^12^ These observations suggest that CagX and CagY are important in the early events mediating *H. pylori* interaction with host cells, which are independent of and additional to the T4SS-mediated translocation of CagA.

The divergence between Cag and Vir proteins is particularly striking for the large CagY protein, which differs in size from other VirB10s by > 100 kDa. The disparity in size is largely attributable to two novel regions of repetitive sequence in CagY, with the second and largest region, CagY_rpt2_, comprising a regular disposition of six repetitive consensus motifs of 5–14 aa, denoted as δ, μ, α, ε, λ, and β.^13^ In the genome-sequenced *H. pylori* strain 26695, the repetitive motifs comprise 74 contiguous segments and span a region of 906 aa, accounting for nearly half the CagY protein.^13^ Flanking this large repetitive region are putative transmembrane domains that potentially delineate a smaller processed form of CagY, which is associated with the T4SS filament assembly.^9,13^ The central repetitive region is further characterised by a regular distribution of cysteine residues, occupying conserved positions in four out of the six repetitive motifs, and an unusual prevalence of lysine and glutamate multiplets. This amino acid composition likely contributes to the stability of post-secretion CagY via the formation of extensive disulphide linkages and electrostatic interactions, respectively.^13^

Underlying the unusual CagY_rpt2_ amino acid composition is extensive repetitive DNA sequence comprising numerous direct DNA repeats.^14^ The repeats are susceptible to in-frame deletion and duplication as a likely consequence of slipped-strand misalignment during DNA replication in a manner independent of RecA.^14^ The resulting contraction and expansion of component motifs in CagY_rpt2_ in addition to polymorphic sequence positions within all motifs have been suggested to contribute to significant phenotypic variation and to be a potential mechanism for evasion of host immune responses.^14^

As the major component of surface-exposed and filament-associated CagY, the large variable CagY_rpt2_ is of significant interest. The conserved repetition of sequence motifs within CagY_rpt2_ is suggestive of a regular repetitive structure that defines CagY function. However, the nature of the putative repeats and the basis for structural and functional tolerance of CagY variation are presently unknown.

Here, we define two predominant repetitive motifs within the CagY_rpt_ region. We determine and compare the secondary structure and stability of isolated repeats with the entire CagY_rpt_ region and demonstrate inter-repeat interactions that allude to their modular assembly in CagY. By cysteine replacement, we show that interactions between isolated repeats can occur both dependently and independently of covalent disulphide linkages and show functional conservation of different CagY_rpt2_ motif arrangements. Finally, we discuss the structural basis for CagY functional conservation as an intrinsic feature of the component repetitive unit.

## Results

### CagY_rpt2_ sequence annotation

**Fig. 1:**
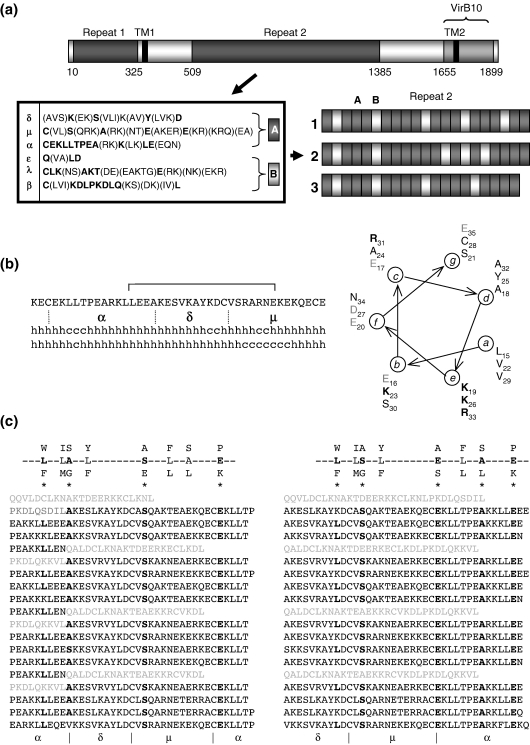
(a) Schematic representation of pre-secretory CagY of the genome-sequenced *H. pylori* strain HP26695 illustrating key domains and motif annotation. Approximate amino acid position of each region is indicated. The C-terminal ‘VirB10’ domain of CagY shares 31% identity (BLAST E value = 3e− 21) with ∼ 55% of the *A. tumefaciens* VirB10 protein. The putative secreted filament-associated form of CagY comprises the large repeat 2 region defined by the two transmembrane (TM) domains. The extensive repetitive sequence of the repeat 2 domain comprises six submotifs (ε, λ, β, δ, μ, and α) invariantly organised into two larger principal repetitive motifs, A and B. Annotation for consensus A (δμα) and B (ελβ) sequence motifs is shown in the inset. Motif organisation within the repeat 2 region is shown for both genome-sequenced *H. pylori* strains HP26695 (1) and J99 (2) and for clinical strain Q121B (3). Expansion/contraction of the CagY_rpt2_ from different strains due to acquisition and loss of component motifs is clearly illustrated. (b) Sequence properties of the CagY_rpt2_ A motif. Prediction of secondary structure (h, helix; c, random coil) representing the consensus of several predictive programs for sequence predominantly comprising the CagY A motif. Submotif sequence annotation is indicated (α, δ, μ). The brace over the peptide primary sequence indicates the 21 residues comprising three consecutive heptad repeats, indicative of coiled-coil structure. Helical wheel representation of the three tandem heptads is shown at the far right, in which residue numbering occurs from the N-terminus of the complete peptide sequence. Note the distribution of hydrophobic residues in *a*/*d* positions and acidic/basic residues (bold/light grey, respectively) in *g*/*e* positions. (c) Contiguous sequence within the entire CagY_rpt2_ region of strain Q121B can also be aligned against the TPR consensus sequence [WLF]-X(2)-[LIM]-[GAS]-X(2)-[YLF]-X(8)-[ASE]-X(3)-[FYL]-X(2)-[ASL]-X(4)-[PKE]. CagY_rpt2_ A motif repeats match the TPR consensus at either 4/8 (left alignment) or 5/8 (right alignment) consensus positions, representing either αλμα or λμα submotifs, respectively, as highlighted in boldface and indicated by an asterisk. Submotif identity (α, δ, μ) is shown beneath the sequence for repetitive motif A only. The sequence of motif B as it occurs interspersed between tandem A motif repeat sequence is shown in faint grey text.

A previous study reported a statistical analysis of CagY_rpt2_ motif composition derived from a single CagY sequence from the genome-sequenced strain 26695. Six repetitive submotifs (termed δ, μ, α, ε, λ, and β) were defined and suggested to be organised into three principal motifs, comprising a combination of three submotifs each [(α, ε, λ), (β, δ, μ), and (α, δ, μ)].^13^ Using the same submotif designation, we reassessed the CagY_rpt2_ motif composition by comparison of 14 full-length CagY sequences presently available in the National Center for Biotechnology Information non-redundant protein sequence database. This revealed an extended consensus sequence for each submotif and, more importantly, suggested a different motif structure from that originally described; when organised as triads of three distinct submotifs each, CagY_rpt2_ can be shown to comprise tandem arrays of a predominant motif repeat (δμα) punctuated at intervals by a second, less abundant motif (ελβ). For brevity, we term these A (δμα) and B (ελβ) (Fig. 1a). Both the 38- to 39-residue A motif and the 31-residue B motif are completely conserved throughout the CagY_rpt2_ region with respect to their submotif composition and are predicted to comprise extensive α-helical secondary structure. Demarcation of motif sequence boundaries by this alternative annotation clearly indicates the modular nature of component repeats and alludes to a regular structural organisation of CagY_rpt2_.

### Motif analysis

Our definition of a simplified repeat structure within CagY_rpt2_ enabled a targeted motif analysis of the repeat region. Internal protein repeats tend to possess regular secondary structure and are known to confer functional and structural versatility to diverse proteins.^15,16^ There are many known classes of repeat, but degeneracy of repeat sequence is common due to divergent evolution and functional specialisation. Consequently, identification of repeats belonging to any particular class is often challenging.^16,17^ In part, this explains why motifs within the CagY sequence have not been reported previously, despite rigorous analysis. Our initial *in silico* analyses proved similarly unenlightening; BLAST/PSI-BLAST searches with the defined CagY A and B repeat sequence failed to identify homologues, and comparison of CagY sequences against motif and pattern databases using an extensive suite of motif discovery tools also failed to recognise known motif signatures. Consequently, guided by motif sequence alignments in REP^17^ and Pfam consensus sequences,^18^ we collated consensus sequence data for known classes of α-helical repeats and examined the defined CagY_rpt2_ A and B motif sequences by manual alignment, in addition to a more general assessment of sequence characteristics.

In addition to predicted α-helical structure, the more abundant A motif in particular has distinct amphipathic character and sequence heptad periodicity, the latter being an established marker for α-helical coiled-coil conformation. Consistent with this, confident predictions of coiled-coil propensity were indicated by both COILS (default settings, window 28, 100% confidence) and MultiCoil (default settings, 52.8% confidence) predictive programs. Coiled-coil sequences are characterised by consecutive heptad repeats. Each repeat of seven residues, denoted *abcdefg*, has characteristic amino acid composition, whereby residues occupying positions *a* and *d* are frequently hydrophobic and those occupying positions *e* and *g* are charged.^19^ The *a*/*d* position residues form a continuous hydrophobic core in the centre of a coiled-coil helical bundle, which is stabilised by electrostatic interactions between *e*/*g* position residues of opposing helices.^19^ A helical wheel plot illustrates this characteristic residue composition of three consecutive heptads within the A motif (Fig. 1b).

Amphipathic α-helices are also characteristic of other α-helical repeats, including the tetratricopeptide repeat (TPR). The TPR is a degenerate 34-residue repeat often present in tandem arrays of 3–16 motifs.^20–22^ Each TPR motif comprises a pair of α-helices (helices A and B) that adopt a helix–turn–helix arrangement, generating a right-handed superhelical shape. Helix A interacts with helix B and helix A′ of an adjacent TPR. TPRs have been identified in diverse proteins with functions ranging from protein transport and folding to transcriptional regulation.^20–22^ We find that the TPR Pfam consensus [WLF]-X(2)-[LIM]-[GAS]-X(2)-[YLF]-X(8)-[ASE]-X(3)-[FYL]-X(2)-[ASL]-X(4)-[PKE]^18^ can be aligned at several different positions in the A motif. The two most credible alignments match either 5/8 positions precisely spanning the A motif (δμα) or 4/8 positions largely comprising (δμ) submotifs flanked on either side by terminal portions of α submotifs (αδμα) (Fig. 1c). Notably, these latter segments are also indicated to comprise TPR segments by REP predictions when no prediction threshold is applied.

Due to the degenerate nature of the TPR sequence, few TPR helices match the consensus at all eight positions. Similarly, although the 38- to 39-aa CagY_rpt2_ A motif is larger than a typical TPR, additional intervening sequence between adjacent TPRs has been reported to extend some TPR-like α-helical segments beyond the 34-aa consensus sequence. No further motif signatures were evident for the A motif and none could convincingly be aligned against the sequence of the B motif. Superficial sequence similarity of TPR helices with coiled coils is such that TPR helices were originally proposed to adopt a type of coiled-coil structure with similar ‘knobs in holes’ packing of side chains from adjacent interacting helices,^23^ possibly explaining the identification of both signatures within the same sequence.

These *in silico* analyses provide the first report of the possible nature of the predominant repetitive motif A subunit and allude to a regular structural assembly of the CagY_rpt2_ potentially mediated by α-helical interactions between adjacent motifs.

### Hetero-oligomeric interactions between CagY_rpt2_ principal motifs

To assess the potential for specific interaction between adjacent and more distant motifs (A with A and/or A with B) in the assembly of CagY_rpt2_, we initially constructed a *cagY* intragene mini-library for assessment of pairwise repeat interactions in the yeast two-hybrid system.

The *cagY* sequence of a clinical isolate, Q121B, was used to design oligonucleotide primers that anneal at multiple conserved sites in the encoded CagY_rpt2_ region (forward and reverse primers to sequence encoding KECEKLL and KLLTPEA of the α motif, respectively, Fig. 1a). Low stringency PCR subsequently enabled amplification of defined fragments ranging from 147 to 2238 bp in size, representing both single and tandem arrays of component A and B motifs along the length of CagY_rpt2_. Amplified fragments were cloned to plasmids pGAD424 (Gal4 activation domain ‘prey’ plasmid) and pGBT9 (Gal4 binding domain ‘bait’ plasmid), and library representation was assessed by restriction enzyme excision of inserts and visualisation of a tight laddering of bands within the expected size range by agarose gel electrophoresis (not shown).

For assessment of CagY fragment interactions, the yeast reporter strain PJ69-4A was co-transformed with bait and prey plasmids expressing low levels of Gal4–CagY fusions to all combinations of CagY fragments. Interactions were indicated by activation of reporter combinations (*HIS3*, *ADE2*, and *lacZ*) allowing direct assessment of the yeast two-hybrid phenotype by the colour of colonies growing on selective media. Consequently, blue yeast colonies (*lacZ* activation) growing on selective media (*HIS3* and *ADE2* activation) were selected for plasmid re-isolation.

**Fig. 2:**
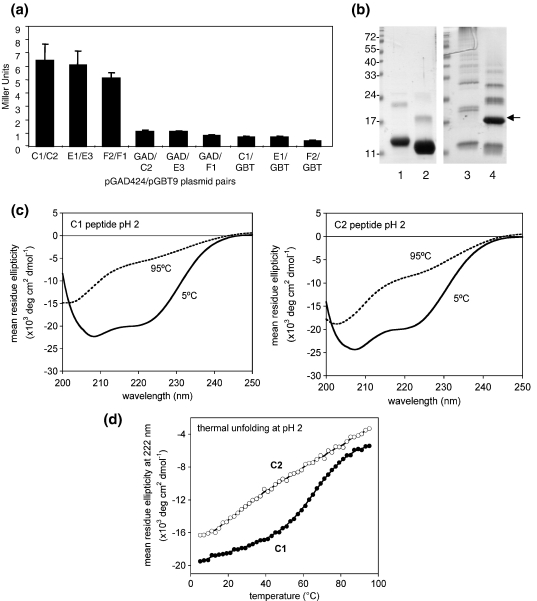
(a) β-Galactosidase liquid assay. Four pairs of interacting CagY_rpt2_ fragments isolated from yeast two-hybrid library screens were assessed by liquid assay and compared to activity of single fusions/empty vector self-activation controls. Interacting pairs C1/C2 (motifs A/BA), E1/E3, and F2/F1 (motifs AA/BA) showed a 5.6- to 12.3-fold increase over self-activation controls. Three independent assays were performed for each interaction (*n* = 3). Error bars represent standard deviation from the mean. (b) Affinity-purified His-tagged CagY-C2 (lanes 2 and 4) and C1 (lanes 1 and 3) fragments representing motifs A and B, respectively, were analysed by reducing (lanes 1 and 2) and non-reducing (lanes 3 and 4) 15% SDS-PAGE. Multimeric forms of both motifs are clearly evident in the absence of reducing agent (lanes 3 and 4), with homodimer predominating for the CagY-C2 protein (arrow). (c) CD spectra show characteristic minima at 208/222 nm, indicating substantial α-helical secondary structure for both C1 and C2 proteins. Notably, both proteins retain significant structure during thermal unfolding (dotted lines). (d) However, in contrast to the non-cooperative transition of the CagY-C2 protein, CagY-C1 demonstrates significant cooperativity during thermal unfolding at low pH as indicated by the sigmoidal curve (transition midpoint at ∼ 70 °C). Samples were analysed in 10 mM sodium acetate (pH 2).

Inserts contained within 10 pairs of interacting plasmids were isolated and sequenced. Accounting for duplication of inserts in the 20 different constructs sequenced, only four different CagY motif fragments representing three different interactions were revealed from the interaction screen, despite the apparent overrepresentation of fragments accounting for the entire CagY_rpt2_ in the mini-library. Representative interacting CagY repetitive motif fragments encoded by pGAD424/pGBT9 constructs C1/C2 (two interactions), E1/E3 (five interactions), and F2/F1 (three interactions) are shown in Table 2. Inserts comprised motif A in both single (A) and double (AA) copy (inserts C2 and F2, respectively), as well as motif A contiguous with motif B (BA) (inserts C1, F1, and E3). In the latter case, C1/F1 inserts differed from the E3 insert in the sequence of the λ submotif, suggesting that the motif fragments were derived from different regions of the CagY_rpt2_ (Table 2). Interacting pairs were subsequently assessed for β-galactosidase activity by liquid assay to gain a more quantitative measure of reporter activity; they showed a 5.6- to 12.3-fold increase over self-activation controls (Fig. 2a), confirming the initial positive yeast two-hybrid growth phenotype.

From these results, interactions were therefore indicated between motif A with another motif A and/or motif B. However, subsequent retransformation of the yeast reporter strain with plasmid combinations pGBT-C2/pGAD-E1 (motifs A/AA) and pGAD-C1/pGBT-E3 (AB/AB) proved negative, indicating that only heterodimeric interactions between isolated motifs A and B are permissible in this system. No direct homodimeric interactions (A–A or B–B) were observed for either motif.

### Homo-oligomeric interactions between CagY_rpt2_ principal motifs

Insert C2 encoding the minimal A motif and its yeast two-hybrid interaction partner C1 (BA motifs) were selected for further analysis in order to validate the yeast two-hybrid observations and explore the contribution of disulphide bonding to motif–motif interactions. Cysteine residues are conserved in 4/6 submotif sequences (Fig. 1a), and their abundance along the length of the CagY_rpt2_ raises the possibility that disulphide bonding is a component of motif assembly. However, as disulphide linkages are unlikely to occur in the reducing environment of the yeast nucleus, the yeast two-hybrid system is not a suitable approach for defining a potential stabilising influence of covalent bonds in motif–motif interactions.

Both C2 and C1 inserts were therefore cloned to the T7 expression vector pET17b for over-expression and purification of soluble recombinant N-terminal His-tagged protein (CagY-C2 and CagY-C1) (Fig. 2b). Initial analysis by reducing and non-reducing 15% sodium dodecyl sulphate/polyacrylamide gel electrophoresis (SDS-PAGE) demonstrated the apparent ability of both recombinant proteins to multimerise. In the absence of reducing agent, prominent homodimeric species were evident for CagY-C2 with additional CagY-C2 oligoforms appearing to increase in molecular mass by the addition of one subunit, approximating the order monomer (< 11 kDa), dimer (∼ 17 kDa), trimer (∼ 24 kDa), and tetramer (∼ 32 kDa) until visualisation of bands diminished after a further three to four higher-order multimers (Fig. 2b, lane 4). Size-exclusion chromatography identified dominant peaks of 8.4, 13.8, and 32.1 kDa, corresponding to monomer, dimer, and trimer/tetramer, respectively, when analysed by SDS-PAGE (not shown). Although evidenced by SDS-PAGE, higher-order oligomers could not be further resolved into individual peaks. The higher molecular mass of trimer/tetramer recorded by gel filtration suggests retardation through the column matrix, indicative of an extended conformation presumably reflecting the non-globular nature of the proteins.

The presence of defined multimeric forms of CagY-C2, which appear to increase in size by one monomer each, suggests that association between monomer subunit motifs is specific, uniform, and stabilised by covalent linkages. Of note, no novel species were observed following mixing of purified CagY-C1 and CagY-C2 proteins at various ratios (data not shown), despite the presence of non-interacting monomer in both samples (Fig. 2b, lanes 3 and 4). This suggests that the hetero-oligomeric interactions observed in the yeast two-hybrid system, although clearly permissible, might be associated with a lower affinity compared to a higher-affinity preferential homo-multimerisation of monomer subunit motifs.

The multimerisation of motifs under non-reducing conditions therefore indicates that covalent disulphide interactions contribute to assembly of isolated motifs, either through intramolecular stabilisation of native conformation or through intermolecular stabilisation of motif interactions. Importantly, neither aggregation nor insolubility of protein, indicative of non-specific disulphide bond formation, is evident, suggesting that multimerisation of discrete monomeric repeat motifs is specific and not a consequence of random associations that might otherwise be expected by atmospheric oxidation.

### Biophysical characterisation of CagY_rpt2_ principal motifs

To assess the conformation and stability of CagY_rpt2_ motifs A and B, we measured circular dichroism (CD) properties of the representative His-tagged proteins CagY-C2 and CagY-C1, respectively. In both cases, the far-UV CD spectrum demonstrated characteristic double minima in the ellipticity at 208 and 222 nm, indicative of substantial α-helical secondary structure as predicted (Fig. 2c). The ratio of [θ]_222_/[θ]_208_ can be taken as a measure of α-helicity, in particular the α-helical supercoiling associated with coiled-coil formation, whereby ratios approaching 1.0 or beyond are indicative of fully folded coiled coils.^24^ Under the conditions used, the [θ]_222_/[θ]_208_ ratio for both CagY-C1 and CagY-C2 proteins was 0.86 and 0.8, respectively (corresponding to 51% and 53% helicity when estimated using the mean residue ellipticity at 222 nm), indicative only of single-stranded α-helical conformation. Consequently, although the amino acid sequence of the CagY-C2 fragment has distinct characteristics of coiled-coil propensity, our CD data does not indicate coiled-coil conformation.

By observing changes in the signal at 222 nm with increasing temperature (5 to 95 °C), CagY-C1 could be shown to undergo a cooperative thermal unfolding transition giving a sigmoidal melting curve with a transition midpoint at ∼ 70 °C (Fig. 2d). The nature of the unfolding curve was shown to be pH dependent as the characteristic sigmoidal melting curve evident at low pH was replaced by a broader non-cooperative transition between pH 5 and pH 7. However, neither helical content nor thermal unfolding showed any concentration-dependent effects, suggesting that the transitions are associated with intramolecular unfolding processes rather than a consequence of intermolecular dissociation of multimers.

In contrast, CagY-C2 demonstrated a broad non-cooperative thermal unfolding transition at low pH with less of an apparent reduction in overall ellipticity than observed for CagY-C1 over the same temperature range (Fig. 2d). This suggests that any unfolding may be relatively localised and that the polypeptide chain is more resistant to global thermal unfolding. No concentration or pH-dependent effects were observed. As single-stranded amphipathic α-helices tend to be unstable in solution, the extreme thermal stability observed for the CagY-C2 protein is therefore a likely consequence of specific conformational properties or the effects of multimerisation. However, as noted, multimerisation of CagY-C2 is not indicated to involve the helical supercoiling associated with coiled-coil conformation.

The observation that the A motif remains stably folded across a range of physical parameters fully supports the discrete modular nature of motifs suggested by the sequence annotation (Fig. 1a). As the predominant motif within the CagY_rpt2_ region, such physical properties would be entirely compatible with the stability and pH resistance presumably required for CagY_rpt2_ to function as a surface-exposed sheath providing mechanical support for the large *cag*-encoded T4SS filament structure.

### Site-directed mutagenesis of CagY-C2

**Fig. 3:**
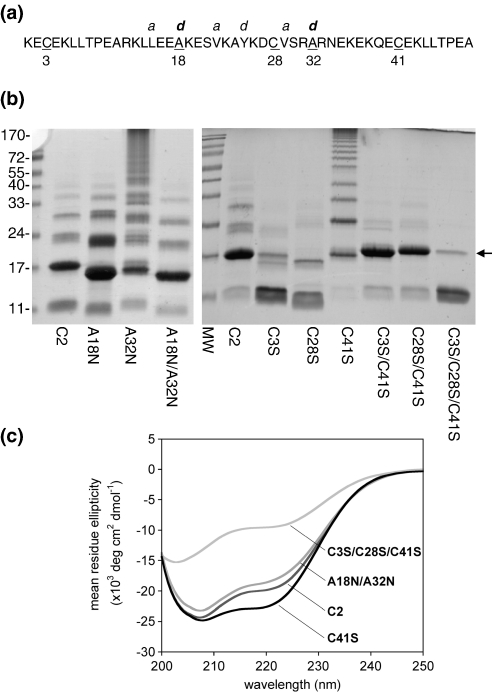
Multimerisation and secondary structure of CagY-C2 substitution mutants. (a) Single, double, and triple CagY-C2 mutants were generated by substitution of either alanine at hydrophobic heptad *d* or multiple cysteine positions (underscore). Substitution at hydrophobic positions had little effect on multimerisation of the C2 protein (b, left panel), suggesting lack of coiled-coil conformation. However, abrogation of multimeric isoforms was evident with the Cys mutants (right panel), although exceptionally, CagY-C2_C41S_ exhibited increased multimeric potential. Notably, homodimer was still evident in the C2_C3S/C28S/C41S_ triple mutant following total abrogation of covalent disulphide interactions (arrow). (c) Comparative CD spectra for CagY-C2 and mutant derivatives demonstrate conservation of α-helical structure (25 μM protein, 5 °C). CD profiles shown in descending order for C2_C3S/C28S/C41S_, C2_A18N/A32N_, C2, and C2_C41S_, respectively.

Our previous experiments suggest that the A motif requires disulphide linkages to stabilise subunit multimerisation and that subunit associations are not a consequence of coiled-coil conformation. To provide more definitive support for these observations, we generated a panel of A motif mutants substituted at either hydrophobic heptad *d* or Cys positions in the CagY-C2 protein. Nine single, double and, triple substitution mutants, CagY-C2_A18N_, C2_A32N_, C2_A18N/A32N_, C2_C3S_, C2_C28S_, C2_C41S_, C2_C3S/C41S_, C2_C28S/C41S_, and C2_C3S/C28S/C41S_, were constructed and assessed for their ability to multimerise as before. Of these, the CagY-C2_A18N_, C2_A32N_, and C2_A18N/A32N_ mutants represent single and double heptad *d* position substitutions (Figs. 1b and 3a), which would be expected to disrupt helical associations mediated by coiled coils. In agreement with the previous CD data, however, none of the hydrophobic substitution mutants were abrogated in their ability to multimerise (Fig. 3b, left panel). Additionally, CD profiles for these mutants were almost identical with the wild-type CagY-C2 protein and showed negligible decreases in helical content (Fig. 3c), further indicating lack of coiled-coil conformation in CagY-C2 multimerisation.

Conversely, Cys–Ser substitution of combinations of the three A motif cysteine residues effectively reduced the ability of CagY-C2 to multimerise in all but one case (Fig. 3b, right panel). The reduced multimerisation observed for the majority of mutants compared to the wild-type CagY-C2 protein is a likely consequence of either loss of stabilising disulphide bonds or loss of local conformation that subsequently promotes non-specific but finite disulphide linkages between structurally defective mutant monomers.

In distinct contrast to the majority of mutants, substitution of Cys41 in CagY-C2_C41S_ significantly enhanced subunit multimerisation to the extent that a limitless laddering of sequential multimers was apparent (Fig. 3b). In terms of thermodynamic stability, this laddering profile may represent the optimum packing arrangement of consecutive α-helical subunits. The estimated helical content of CagY-C2_C41S_ using the mean residue ellipticity at 222 nm shows a modest increase compared to CagY-C2 (Fig. 3c), suggesting that multimerisation of CagY-C2_C41S_ may arise from a further structural consolidation of the folded monomers consistent with slightly improved helical packing. Potentially, the Cys41Ser substitution relieves the effects of unfavourable disulphide linkage mediated by Cys41 in the A motif fragment, which otherwise predisposes towards the alternative finite subunit associations seen in the wild-type CagY-C2 protein.

Low estimated helical content of the CagY-C2_C3S/C28S/C41S_ triple mutant (25%), however, is consistent with significant loss of local conformation and highlights a critical contribution of the conserved α and μ submotif Cys residues to secondary, tertiary, and quaternary structure. However, despite this, sufficient structure is evidently still present to mediate vestigial CagY-C2_C3S/C28S/C41S_ dimerisation (Fig. 3b, arrow), indicating that discrete A motif monomer interactions can occur in the absence of disulphide linkages.

Taken together, these results show that disulphide bonding is important for both the stability and the homomeric assembly of isolated A motifs, that the α and μ submotifs are important for these interactions, and that an optimum arrangement of A motifs with respect to each other will accommodate the association of an apparently limitless number of motifs. This latter observation is particularly relevant in the context of the CagY_rpt2_ region where A motifs vary widely in number along the length of the repeat 2 region and in equivalent regions of different CagY proteins (Fig. 1a). The lack of demonstrable coiled-coil conformation also directs attention towards other amphipathic α-helical repeat families for the identity of the A repeat module.

### Biophysical characterisation of entire CagY_rpt2_ regions

Although CagY_rpt2_ A motifs are indicated to be modular and, therefore, individually well folded and structurally discrete, isolated motif fragments might demonstrate different biochemical/biophysical properties outside of the context of the CagY_rpt2_ region. Therefore, we sought to determine to what extent our observations with CagY-C2 and its mutant derivatives reflected properties of the CagY_rpt2_ region as a whole.

**Fig. 4:**
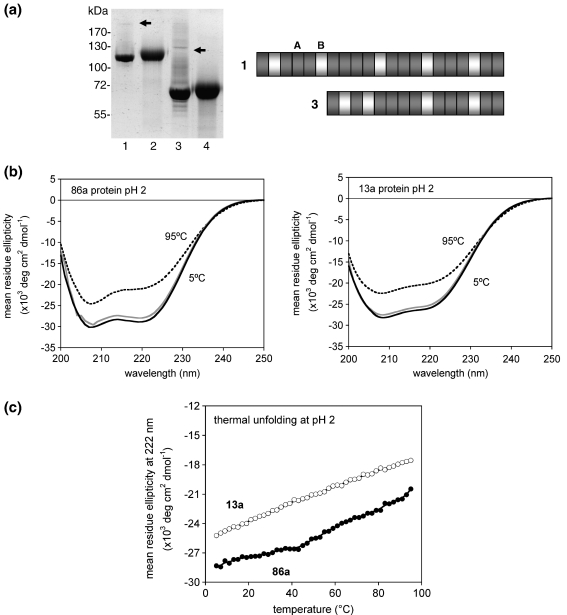
Analysis of the entire CagY_rpt2_ region. (a) Full-length CagY_rpt2_ regions comprising different motif organisations from *H. pylori* strains 13A and Q86A were affinity purified and resolved by SDS-PAGE (lanes 1 and 2 and lanes 3 and 4, respectively). Samples were run under both non-reducing (lanes 1 and 3) and reducing conditions (lanes 2 and 4). The presence of reducing agent does not significantly affect the migration of either protein. Notably, however, possible dimeric species are observed for both proteins in the absence of the reducing agent (arrows). Motif composition and organisation for both are illustrated in the accompanying cartoon (equivalent labels). (b) Both Q86A (left panel) and 13A (right panel) CagY_rpt2_ regions demonstrated highly α-helical secondary structure, which proved resistant to thermal denaturation (dotted line), as the component CagY-C2 A motif. Notably, CD profiles remained unchanged in the presence of 15 mM DTT (grey line). (c) As observed for CagY-C2, minimal thermal unfolding of both proteins was non-cooperative.

Due to difficulties expressing Q121B CagY_rpt2_ at sufficiently high levels, we studied the CagY_rpt2_ of two further strains, Q86A and 13A. Soluble protein was obtained for both and purified to homogeneity (Fig. 4a). Advantageously, both CagY_rpt2_ regions comprise similar motif sequence to Q121B but different motif compositions (Fig. 4a). Additionally, these two proteins represent the minimum (86a) and near-maximum (13a) permissible size observed for the CagY_rpt2_ region, supporting previous observations that the total length of the CagY_rpt2_ is conserved within a defined size range.^14^

Size-exclusion chromatography showed both CagY_rpt2_ proteins to migrate as a single species with molecular mass of 159.7 kDa (13A) and 92.6 kDa (Q86A) (not shown), which differs significantly from both the predicted values of 92.7 and 67.5 kDa, respectively, and the observed migration of these proteins in SDS-PAGE gels (∼ 110 and 68 kDa, respectively, Fig. 4a). In agreement with the column fractionation of trimeric/tetrameric CagY-C2 (not shown), the retarded migration of these proteins suggests that CagY_rpt2_ is non-globular and likely adopts an extended conformation. Notably, neither CagY_rpt2_ region showed any significant difference in migration when analysed in the presence or absence of 15 mM dithiothreitol (DTT) (Fig. 4a), suggesting that disulphide bonds are either buried in the protein and not accessible to reducing agent or not a component of intramolecular CagY_rpt2_ assembly. For the same reasons, CD spectra obtained for both proteins in the presence of 15 mM DTT were also virtually identical with wild-type spectra (Fig. 4b). Of note, non-reducing gels showed vestigial amounts of a possible dimeric species for both CagY_rpt2_ regions (Fig. 4a, arrows), indicating a potential for intermolecular CagY_rpt2_ interactions mediated by disulphide linkages.

Estimated helical content based on the mean residue ellipticity at 222 nm was high, but only slightly different for both Q86A (72%) and 13A (64%) CagY_rpt2_ regions, reflecting a modest difference in stability arising from the different ratio of A and B motifs in each (Fig. 4b). Both regions were equally resistant to denaturation (Fig. 4c), retaining equivalent levels of helical content across a range of temperature (5–100 °C) and pH (2–7) and showing no dependence upon concentration (5–50 μM) for structure or stability. These properties are equivalent to those observed for the component A and B motif subunits, demonstrating the efficacy of studying isolated repeats and further alluding to the modular organisation of motifs within CagY_rpt2_. Entire CagY_rpt2_ regions are therefore shown to be extremely structurally stable within a broad range of physical conditions, consistent with structural preservation of the exposed protein within the fluctuating environment of the *H. pylori* gastric niche.

### Functional characterisation of different CagY_rpt2_ regions

The previous experiments confirmed that structural integrity was maintained for both Q86A and 13A CagY_rpt2_ regions despite a substantial difference in motif composition and organisation (Fig. 4a). However, if A motifs are indeed modular as the experimental evidence suggests, then it might be expected that both structure and function of the CagY_rpt2_ region would be preserved following motif gain and loss. Therefore, we next sought to determine if the different CagY_rpt2_ regions affected a fundamental function of CagY.

CagY is essential for the functionality of the T4SS of *H. pylori* since a *cagY* deletion mutant is unable to translocate the CagA effector protein to host cells.^8,11^ Upon delivery to the inner side of the host plasma membrane, CagA becomes tyrosine phosphorylated by host kinases,^5–7^ providing the basis for a convenient assay of translocated protein as a measure of the functional competence of the T4SS.

As illustrated (Fig. 4a), Q86A CagY_rpt2_ is severely truncated with respect to 13A, having apparently lost multiple complete amino-terminal A and B motifs without interruption to the *cagY* reading frame or subsequent translation of the protein. We therefore assessed both CagA secretion and translocation in the background of these strains using an *in vitro* infection model. Each strain was co-cultured with monolayers of the AGS gastric epithelial cell line, and supernatants tested for the presence of CagA and infected AGS cells were lysed for detection of phosphorylated CagA.

**Fig. 5:**
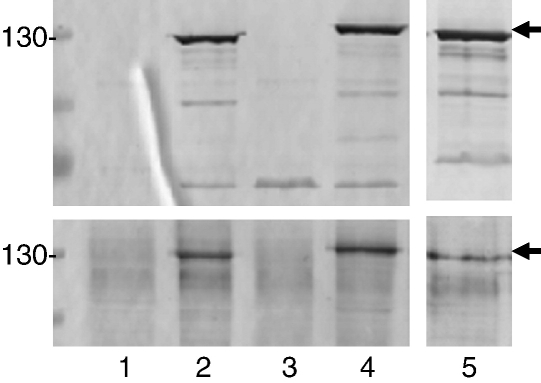
*H. pylori* strains 13A and Q86A, each comprising CagY with a different repeat 2 region, were assessed for their ability to translocate CagA into epithelial cells in a type IV secretion-dependent manner. The top panel shows immunodetection of ∼ 130 kDa CagA secreted into culture medium by *H. pylori* strains 60190(*cag*+), Tx30a(*cag*−), 13A, and Q86A in lanes 2, 3, 4, and 5, respectively. CagA size variation is similarly due to deletion and duplication of repetitive motifs. Lane 1 shows absence of CagA in uninfected supernatants. The bottom panel shows immunodetection of tyrosine-phosphorylated CagA following translocation to and modification in host cells in a type IV secretion-dependent manner. Lane designations are the same as for the top panel.

Both Q86A and 13A strains were shown to be equally competent for secretion and delivery of CagA to host cells (Fig. 5, lanes 4 and 5, respectively) despite the large disparity in CagY_rpt2_ motif composition. Consequently, CagY_rpt2_ is shown to exhibit remarkable structural tolerance for deletion or duplication of component motifs, supporting previous data that individual motifs within the CagY_rpt2_ comprise discrete modular structural domains that can be inserted or deleted without compromising the global CagY_rpt2_ structure or function.

## Discussion

In this study, we have investigated the large enigmatic repeat region of the secreted virulence-associated protein CagY. We present a novel sequence annotation for the CagY_rpt2_ region that defines two principal repetitive motifs, termed A and B. Characteristically, tandem arrays of one to six A motifs are flanked by single B motifs along the entire length of CagY_rpt2_ (Fig. 1a). The motif annotation clearly shows that duplication and deletion of whole motif segments result in strain-specific CagY motif content and organisation without compromising the underlying modular submotif composition; both principal motifs (A and B) comprise three distinct submotifs each, which remain invariant in their order with respect to each other. Furthermore, although individual submotifs have multiple polymorphic positions, variant residues are largely conserved with respect to the size, charge, or hydrophobicity and are flanked by strictly conserved positions (Fig. 1a). These features strongly infer preservation of an underlying conserved structure defined by each principal motif. Consequently, CagY_rpt2_ is indicated to have a modular structural organisation comprising repetition of a single predominant repeat unit (A motif repeat), the number of which in any particular array being delimited by a single flanking B motif.

Biophysical analysis of representative CagY_rpt2_ A and B motif fragments (CagY-C2 and CagY-C1, respectively) initially isolated in a yeast two-hybrid interaction screen confirms secondary structure predictions that both motifs comprise significant α-helical structure. Both helical repeats also demonstrate remarkable thermal and pH stability and suggest that isolated repeat modules are individually well folded (Fig. 2c and d). The modular nature of the A motif in particular is further reinforced by demonstration of stable and specific homo-multimerisation of recombinant protein, which indicates a capacity for interactions between adjacent A motif repeats in the assembly of the CagY_rpt2_ structure (Fig. 2b). Additional support for modular A motif structure is gained from the observation that discrete repeats can be deleted or duplicated without obvious detrimental effects to CagY structure or function, since CagY_rpt2_ regions comprising very different motif organisations remain well folded (Fig. 4b and c) and fully competent for translocation of CagA to host cells (Fig. 5).

Collectively, these observations are characteristic of α-helical repeat arrays. Repeat proteins comprise structurally identical motifs arranged in tandem arrays. The repeat regions tend to adopt an elongated shape that forms a large binding surface serving as a scaffold for multiple protein–protein interactions in diverse cellular pathways.^15–17^ Several different families of 20- to 40-aa α-helical repeats, comprising one to three component α-helices, have been defined. Local interactions between constituent α-helices and α-helices of adjacent repeats produce an integrated superhelical structural assembly.^15–17^

*In silico* analyses of the defined CagY_rpt2_ A and B motif sequences identify signatures and the characteristic residue composition of two ubiquitous α-helical repeats in the sequence of the A motif: coiled coils and the TPR. Coiled coils are well-characterised and intensively studied interaction motifs. The sequence requirements and predictable manner by which coiled-coil α-helices associate make them ideal structures for the study of protein folding, not least because coiled-coil conformation can be readily identified and assessed by biochemical/biophysical approaches.^19,24^ However, this is not the case for other common α-helical repeat motifs such as TPRs where the identity and nature of the repeat can only be confirmed by structural solution of the protein or domain in which it is a component. Therefore, using appropriate approaches for analysis of coiled-coil helices, we show that of the two α-helical repeat families presented as candidate structures of the CagY_rpt2_ A motif, coiled-coil conformation can largely be dismissed; the CD profile of the CagY-C2 A motif is not characteristic of helical supercoiling (Fig. 2c), and mutation of putative helical interface hydrophobic residues does not abolish multimerisation (Fig. 3b).

Conversely, however, much of our data remain consistent with known characteristics of TPR arrays. The TPR is a 34-residue repeat often present in tandem arrays of 3–16 motifs. All TPR arrays for which structures have been solved to date are shown to be terminally flanked (‘capped’) by a non-TPR solvating α-helix^20–22,25,26^; the α-helical CagY A motif occurs in tandem arrays of 1–6 motifs terminally flanked by a single α-helical B motif (Fig. 1a). The CagY_rpt2_ motif organisation is therefore reminiscent of a novel arrangement of tandem TPR arrays, whereby each array differs in the number of component A motifs and is invariantly capped by a single B motif before the start of the next array. Although, to our knowledge, an equivalent arrangement of tandem TPR or TPR-like arrays has not been described, it may reflect functional/structural specialisation of CagY or the susceptibility of the CagY_rpt2_ to undergo extensive contraction and expansion of component motifs^14^; multiple copies of the B motif would ensure that essential putative solvating helices were not lost through frequent recombination.

Beyond organisational similarities, the CagY_rpt2_ A motif fits 4/8 or 5/8 consensus TPR positions depending upon which submotif groupings are considered in the alignment (Fig. 1c). However, since A motifs occur in tandem arrays, either putative TPR arrangement can feasibly be accommodated. The CagY_rpt2_ B motif sequence does not convincingly fit with any α-helical repeat consensus that we can identify; however, it does have similar residue composition to the A motif, which is, again, reminiscent of equivalent properties of the typical TPR solvating helix.^20–22,25,26^

TPRs comprise two α-helical domains that are defined by the consensus residues 4, 7, 8, and 11 (helix A) and 20, 24, 27, and 32 (helix B) as denoted in Fig. 1c. Helix A interacts with helix B, generating the characteristic helix–turn–helix TPR fold, as well as with helix A′ of an adjacent TPR. As such, assembly of the regular folded TPR structure involves interactions between adjacent repeats. Consistent with this, our data show that isolated A motifs expressed as recombinant protein appear well folded by CD, suggesting local conformation, and are shown to multimerise, indicating uniform interaction of a repetitive modular structural unit.

Although the A motif differs in size to the typical 34-aa TPR motif composition, there is a precedent for divergence of motif structure in other *H. pylori* proteins. Members of the *Helicobacter c*ysteine-rich *p*rotein (Hcp) family, HcpC^27^ and HcpB,^28^ are β-lactamases additionally involved in the inflammatory response coincident with *H. pylori* infection.^29^ Both HcpB and HcpC comprise tandem repeats of a 36-aa disulphide-bridged α/α repeat motif that belongs to the SEL1 subfamily of TPR proteins.^18,27–29^ Although structurally similar to the TPR, the additional two amino acids in the SEL1 repeats extend the short loop between antiparallel α-helices of the unit motif, resulting in a different helix packing angle compared to typical TPR motifs. The 38- to 39-aa CagY_rpt2_ A motif might similarly incorporate an extended loop region presenting a novel repeat conformation.

Notably, the SEL1 family repeats are also distinguished by covalent bonding between motifs. Although cysteine residues feature in other repeat proteins, to our knowledge, intramolecular disulphide bonds have been reported in only three to date, which includes HcpB and HcpC.^28–30^ The regular disposition of Cys residues in both A and B motifs presents the prospect of extensive disulphide bonding. We show that substitution of Cys residues profoundly affects multimerisation of isolated A motifs outside of the context of the CagY_rpt2_ in a manner entirely consistent with the abrogation of disulphide linkages. It is presently unclear, however, whether covalent interactions stabilise either intramolecular motif interactions in the global structure of the CagY_rpt2_ or intermolecular motif interactions that facilitate assembly of CagY_rpt2_ subunits into the filament sheath. The extreme stability of the CagY_rpt2_ region and the observation of possible CagY_rpt2_ dimers lend support to both scenarios. The possibility that disulphide linkages are peculiar to interactions between isolated monomeric A motifs also cannot be excluded, although as we observe, the tendency of disulphide bonds to stabilise folded rather than unstructured proteins^31,32^ is further evidence that the CagY-C2 protein comprises native structure reflective of a modular α-helical repeat.

The dependence of isolated motifs for stabilisation by disulphide linkages and the complexity and extent of sequence repetition in *cagY* essentially preclude a more comprehensive mutagenesis study of the CagY_rpt2_ to fully address the nature of component motifs and their associations. However, our data demonstrate that isolated motifs fold as stable α-helices, which are competent for a range of homotypic interactions, consistent with CagY_rpt2_ comprising a succession of discrete and modular structural domains mediating regular assembly. These experimental observations, together with identification of consensus TPR sequence, tandem repetition of motifs, and punctuation of repetitive arrays with putative solvating helices, provide persuasive evidence for a novel arrangement of modular TPR-like arrays within the CagY_rpt2._

In summary, our findings provide rational explanation for the diversity and unusual sequence features of CagY variants and reveal CagY structural features that are compatible with its observed functional role as a mechanically protective filament sheath.^9^ Future studies should now be directed towards structural solution of component CagY repeat modules, AB (δμαελβ), AAB (δμαδμαελβ), or the entire CagY_rpt2_ and detailed examination of CagY_rpt2_-mediated protein–protein interactions in the assembly and function of the *cag* T4SS.

## Materials and Methods

### Yeast and bacterial strains, plasmids, and growth conditions

Plasmids and bacterial strains are listed in Table 1. *H. pylori* clinical strains Q121B and Q86B were isolated from dyspeptic patients with evidence of duodenal ulcer. All *H. pylori* strains were grown on blood agar plates (Oxoid, Basingstoke, UK) in a microaerobic environment for three passages prior to extraction of genomic DNA or subsequent inoculation to F12-HAM media (Sigma, Poole, UK). *Escherichia coli* strains were grown at 37 °C in Luria broth or agar supplemented with ampicillin (50–100 μg ml^− 1^) as required. *Saccharomyces cerevisiae* strain PJ69-4A was grown at 30 °C and maintained in complete SC medium supplemented with 2% glucose (w/v).

### Protein sequence analysis

Predictions of secondary structure were performed using GOR, HNN, Jpred, and PSIpred programs, accessible through the ExPASy web site.† Coiled-coil predictions were performed using COILS‡ and MultiCoil.§ Motif analyses employed ScanProsite,∥ Pfam,¶ REP,^a^ and the GenomeNet suite^b^ for database and motif library searches. Existing CagY sequences were retrieved from the National Center for Biotechnology Information database^c^ from where BLASTP/PSI-BLAST searches were also performed.

### Yeast two-hybrid *cagY* intragene library construction

The entire *cagY* gene was amplified from genomic DNA of *H. pylori* strains Q121B, Q86A, and 13A, using the Expand High Fidelity PCR Kit (Roche) with primers 5′-GGAATTCATGAATGAAGAAAACGATAAACT-3′ and 5′-GGAATTCTCAATTGCCACCTTTGG-3′ according to kit recommendations. Amplification employed 10 cycles of 94 °C/2 min, 53 °C/30 s, and 68 °C/4 min, followed by an additional 17 cycles differing only by inclusion of a 5-s incremental increase in extension time with each subsequent cycle.

Amplified *cagY* products were purified by gel extraction and cloned to pGEMT-Easy (Promega, Southampton, UK) and sequenced (Geneservice Ltd., Cambridge, UK). *cagY* sequences of strains Q121B, 13A, and Q86A have been assigned accession numbers AM779567, AM779568, and AM779566, respectively, and deposited in GenBank. Based on the sequence information for the Q121B *cagY* gene, forward and reverse primers 5′-GGAATTCGGTAAAGAATGCGAGAAATTGCTCA-3′ and 5′-CGGAATTCTTACGCTTCAGGCGTGAGCAATTT-3′, respectively, both of which anneal at multiple locations, were designed to amplify repeat motifs of varying size and number from within the repeat 2 region of *cagY*. Cycling conditions (30 cycles of 94 °C/45 s, 57 °C/45 s, and 72 °C/30 s) were optimised to obtain a distribution of fragments within the size range 147–2238 bp using *Taq* DNA polymerase (New England Biolabs, Hitchin, UK). Purified fragments (Qiagen Ltd., Crawley, UK) were digested with *Eco*RI and ligated directly to *Eco*RI-digested/dephosphorylated pGAD424 or pGBT9 yeast two-hybrid vectors. Multiple ligations were transformed into *E. coli* XLI-Blue cells. Colonies (*n* = 300–500, representing a > 10-fold overrepresentation of any particular fragment) were recovered from each of two plates by washing into 2.5 mL L broth. Resuspended cells were diluted into 50 mL L broth (Amp^50^) and incubated for 14 h prior to harvest and plasmid extraction. HindIII digest of representative plasmid aliquots showed tight laddering of inserts within the intended size range.

### Yeast two-hybrid interaction screen

Twenty microlitres of both pGAD424 and pGBT9 library constructs were co-transformed into yeast strain PJ69-4A in triplicate, using the high-efficiency lithium acetate transformation procedure.^37^ PJ69-4A contains three separate reporter genes (*HIS3*, *ADE2*, and *lacZ*), each under the independent control of three different *GAL4* promoters (*GAL1*, *GAL2*, and *GAL7*) that provide a high level of sensitivity with respect to detecting weak interaction coupled with a low background of false positives.^35^ Co-transformants were initially selected for the plasmid-encoded markers by plating onto SC minus Trp and Leu (MUHA plates) and then replica plating onto SC minus Trp, Leu, and Ade to select for the *ADE2* reporter (MUH plates); SC minus Trp, Leu, and His (MUA plates) to select for the *HIS3* reporter; and SC minus Trp, Leu, and His plus X-Gal (MUAX plates) to select for activation of the *HIS3*/*lacZ* reporters. Initially, 10 well-isolated blue colonies from MUAX plates were selected at random and streaked onto fresh MUA plates. Interacting pGAD424 and pGBT9 construct pairs were subsequently isolated from the parent yeast strain using the Zymoprep yeast plasmid miniprep kit (Zymo Research, Orange, CA), individually transformed to *E. coli* XLI-Blue and plasmid extracted for sequencing of inserts (Geneservice Ltd.). Isolated pGAD424 and pGBT9 construct pairs were subsequently retransformed to PJ69-4A for confirmation of the selective growth phenotype. Activation of the *lacZ* reporter was assessed by quantification of β-galactosidase activity in PJ69-4A cell extracts using *o*-nitrophenyl-β-d-galactopyranoside as substrate.^38^

### Protein expression, purification, and analysis

Constructs pGBT9-*cagY* C2 and pGAD424-*cagY* C1 (Table 2) were used as template for PCR with primers 5′-GAAGATCTCATATGCATCATCATCATCATCACGGTAAAGAATGCGAGAAATTG-3′ and 5′-CGGGATCCTTACGCTTCAGGCGTGAGTAA-3′ (standard three-stage, 25-cycle PCR, annealing at 60 °C) for *Nde*I/*Bam*HI cloning into pET17b. Forward and reverse primers 5′-GAAGATCTCATATGCATCATCATCATCATCACGGTCTAGCTGATATGAGCGTCAAGGC-3′ and 5′-CGGAATTCTCAATCGCTCAAACCATCCAAAC-3′ were similarly used to amplify the region encoding the entire CagY_rpt2_ from strains Q121B, 86A, and 13A. Fragments were cloned to pET17b as before. Expression of recombinant 6His-tagged proteins was induced with 1 mM IPTG in 500 mL Luria broth for 3 h prior to harvest. Bacterial pellets resuspended in 25 mL Tris–Cl buffer (20 mM Tris and 200 mM NaCl, pH 8.0) were disrupted in a French pressure cell, and resulting lysates were clarified by centrifugation and 0.45 μM filtration prior to affinity purification using Talon resin (BD Biosciences, Oxford, UK). Proteins were eluted in 300 mM imidazole, and fractions were concentrated and buffer was exchanged into 10 mM sodium acetate (pH 5.0 or pH 7) using Vivaspin centrifugal concentrators (Sartorius Ltd., Epsom, UK). Protein concentrations were determined using Coomassie Plus Protein Assay Reagent (Perbio Science Ltd., Northumberland, UK). Purified His-tagged proteins were initially analysed by both reducing and non-reducing 15% SDS-PAGE.

### Site-directed mutagenesis

Site-directed mutagenesis of the *cagY* C2 repeat sequence was performed using the QuikChange II Site-Directed Mutagenesis Kit (Stratagene) using double-stranded pET17b constructs or subsequently mutated vector as template. Complimentary mutagenesis oligonucleotide pairs incorporating single amino acid substitutions used the following sense oligonucleotides: 5′-AGCGAGAAAATTATTAGAAGAAAACAAAGAGAGCGTTAAGGCTTAC-3′ (pCBS8), 5′-TTACAAAGACTGCGTTTCAAGAAACAGGAATGAAAAAGAGAAACAAG-3′ (pCBS9 and pCBS10), 5′-CATCATCACGGTAAAGAAAGCGAGAAATTGCTCACGCC-3′ (pCBS11), 5′-CGTTAAGGCTTACAAAGACAGCGTTTCAAGAGCTAGGAATG-3′ (pCBS12 and pCBS16), and 5′-CAAAAAGAGAAACAAGAAAGCGAGAAATTACTCACGCCTG-3′ (pCBS13, pCBS14, pCBS15, and pCBS16). In all cases, antisense oligonucleotides for each mutagenesis experiment were the reverse compliment of the sense oligonucleotides listed above. Mutated plasmid was generated by temperature cycling (1 cycle of 95 °C, 30 s, followed by 16 cycles of 95 °C, 30 s; 55 °C, 1 min; and 68 °C, 3 min 30 s) in the presence of the high-fidelity *Pfu* DNA polymerase. One millilitre of the synthesised products was transformed into competent *E. coli* XL1-Blue cells, and ampicillin-resistant transformants were randomly selected and inoculated to overnight L-broth cultures for preparation of plasmid (Qiagen Ltd.). Correct incorporation of each mutation was assessed by DNA sequencing. Mutated plasmid was transformed to BL21(DE3)pLysS for over-expression of recombinant His-tagged protein.

### CD

CD measurements were performed on an Applied Photophysics Pi-Star-180 Spectrophotometer. The temperature was regulated using a Neslab RTE-300 circulating programmable water bath and a thermoelectric temperature controller (Melcor). CD spectra were recorded at 5 or 95 °C using a 1-mm quartz cuvette. Protein samples were prepared at concentrations between 0.250 and 50 μM. The secondary structure was studied at pH 7, 5, and 2 using 10 mm sodium phosphate (pH 7), 10 mm sodium acetate (pH 5), or 10 mm HCl (pH 2) as the respective buffering salt. Spectra were recorded from 200 to 260 nm and are the averages of three to five scans, with the appropriate background buffer spectrum subtracted. CD measurements were converted into mean residue ellipticity [θ], using the formula:

$$\left[\theta ]=\theta_{\text{obs}}/(10\times l\times c\times n\right)$$

where θ_obs_ is the observed ellipticity in millidegrees, *l* is the optical path length in centimetres, *c* is molar protein concentration, and *n* is the number of peptide bonds. Thermal denaturation curves were recorded over the temperature range 5–95 °C using a 25-μM protein solution in a 1-mm quartz cuvette. Single-wavelength data were recorded at 222 nm over a single accumulation. The sample was required to reach thermal equilibrium at each temperature for a period of at least 30 s with a tolerance of ± 0.2 °C before recording each data point. Ellipticity data were corrected to mean residue ellipticity using the formula above. Estimates of the percentage of helicity were made using the mean residue ellipticity at 222 nm, as described by Chen *et al.*^39^ using the formula:

$$\%\;\text{Helix}=({\left[\theta \right]}_{\text{obs.222}}\times 100)/[-39,500\times (1-2.57/\text{L})]$$

where [θ]_obs.222_ is the observed mean residue ellipticity at 222 nm and *L* is the number of peptide bonds present.

### Size-exclusion chromatography

Pooled fractions (10 mL) of Talon affinity-purified His-tagged CagY-C2 or mutant derivatives were further characterised by size-exclusion chromatography. A 26/60 Superdex 200 column (GE Healthcare) was equilibrated in 20 mM Tris (pH 8.0) and 200 mM NaCl prior to sample loading and was subsequently run at 2 mL/min, collecting 10-mL fractions. The column was calibrated with known standards under equivalent conditions to produce a calibration curve and, therefore, estimates of molecular weight for fractionated peaks (BioRad, Hemel Hempstead, UK). Pooled elution fractions were concentrated and exchanged into 10 mM sodium acetate (pH 5.0 or 7.0) as before for subsequent biochemical and biophysical analyses.

### Bacterial co-culture and CagA translocation assay

AGS human gastric epithelial cells were seeded into 10 mL F12 Ham media in 25-cm^2^ flasks (1 × 10^6^ cells/flask) and grown at 37 °C, 5% CO_2_, until almost confluent. *H. pylori* strains were harvested from 24- to 48-h blood agar plates into F12 Ham medium; OD_550_ (optical density at 550 nm) was determined, and cell densities were adjusted to OD_550_ = 0.1 before addition to AGS cell monolayers (5 mL/flask; multiplicity of infection, ∼ 100). AGS cells were co-cultured with *H. pylori* for 6 h at 37 °C, 5% CO_2_. Infected monolayers were washed three times with phosphate-buffered saline (PBS), and then cells were scraped from the flasks into 5 mL PBS containing 1 mmol/L sodium vanadate. Cell suspensions were centrifuged at 1000*g* for 10 min, and pellets were resuspended in 80 μL PBS/sodium vanadate and 20 μL 5× sample loading buffer. The samples were boiled for 5 min and analysed by 10% SDS-PAGE and immunoblotting using anti-CagA and anti-phosphotyrosine monoclonal antibodies. Blots were developed with the addition of SigmaFAST 5-bromo-4-chloro-3-indolyl phosphate/Nitro blue tetrazolium substrate (Sigma) following incubation with anti-mouse alkaline phosphatase-conjugated secondary antibodies (Sigma).

## Figures and Tables

**Table 1:** Strains and plasmids

	Relevant genotype and/or description	Source
Strains		
*H. pylori* Q121B, Q86A	*cag*PAI+ strains isolated from patients attending upper endoscopy clinic at the Queen's Medical Centre, Nottingham, UK	This study
*H. pylori* 13A	*cag* PAI+ strains isolated in the Netherlands	Ref. 33
*H. pylori* 60190	Wild type (ATCC 49503); *cag* PAI+	Ref. 34
*H. pylori* TX30a	Wild type (ATCC 51932); *cag* PAI−	Ref. 34
*E. coli* XLI-Blue	*recA1 endA1 gyrA96 thi-1 hsdR17 supE44 relA1 lac* [F′ *proAB lacl*q*Z*Δ*M15* Tn*10* (TetR)]c	Stratagene
*E. coli* BL21(DE3)pLysS	F-*ompT hsdS*B (rB-mB-) *gal dcm* (DE3) pLysS (CmR)	Novagen
*S. cerevisiae* PJ69-4A	*MAT*α *trp1–901 leu2–3112 ura3–52 his3–200 gal4*Δ *gal80*Δ *LYS2∷GAL1–HIS3 GAL2–ADE2 met2∷GAL7–lacZ*	Ref. 35
Plasmids		
pGAD424	*ori*ColE1 *ori*2μ LEU1 P_ADH_∷GAL4′ activator domain∷MCS Ap^R^	Ref. 36
pGBT9	*ori*ColE1 *ori*2μ TRP1 P_ADH_∷GAL4′ binding domain∷MCS Ap^R^	Ref. 36
pET17b	T7 expression vector	Novagen
pGEM-TEasy	High copy number cloning vector	Promega
pCBS1	pGEM-*cagY* (full-length gene from strain Q121B)	This study
pCBS2	pGEM-*cagY* (full-length gene from strain Q86A)	This study
pCBS3	pGEM-*cagY* (full-length gene from strain 13A)	This study
pCBS4	pGAD424-*cagY* C1 encoding 80-residue repeat motif (B)	This study
pCBS5	pGBT9-*cagY* C2 encoding 49-residue repeat motif (A)	This study
pCBS6	pET17b-*cagY* C1	This study
pCBS7	pET17b-*cagY* C2	This study
pCBS8	pET17b-*cagY* C2 (A18N)	This study
pCBS9	pET17b-*cagY* C2 (A32N)	This study
pCBS10	pET17b-*cagY* C2 (A18N/A32N)	This study
pCBS11	pET17b-*cagY* C2 (C3S)	This study
pCBS12	pET17b-*cagY* C2 (C28S)	This study
pCBS13	pET17b-*cagY* C2 (C41S)	This study
pCBS14	pET17b-*cagY* C2 (C3S/C41S)	This study
pCBS15	pET17b-*cagY* C2 (C28S/C41S)	This study
pCBS16	pET17b-*cagY* C2 (C3S/C28S/C41S)	This study
pCBS17	pET17b-*cagY* Q86A CagY_rpt2_	This study
pCBS18	pET17b-*cagY* 13A CagY_rpt2_	This study

**Table 2:**
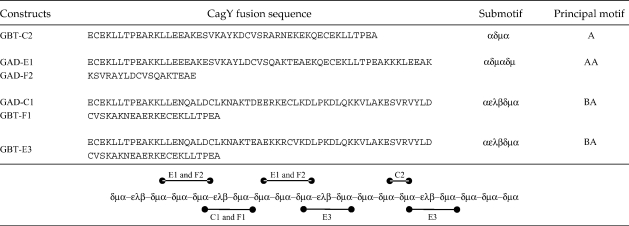
Sequence and motif annotation of representative inserts isolated from four randomly selected interacting yeast two-hybrid constructs

Representative interacting pairs were C2/C1 (motif A with motifs BA), E1/E3 (motifs AA with motifs BA), and F2/F1 (motifs AA with motifs BA). Interacting motif fragments map to 502 aa of virtually contiguous sequence central to the CagY_rpt2_ region. Motif annotation beneath the table illustrates the location of the interacting segments within this repetitive region from parent strain Q121B (spanned by bars). Motif B comprising (ελβ) submotifs is shown in boldface to highlight the disposition of A and B motifs.

## Footnotes

† http://ca.expasy.org/tools/

‡ http://www.ch.embnet.org/software/COILS_form.html

§ http://groups.csail.mit.edu/cb/multicoil/cgi-bin/multicoil.cgi

∥ http://ca.expasy.org/tools/scanprosite/

¶ http://www.sanger.ac.uk/Software/Pfam

a http://www.embl-heidelberg.de/%7Eandrade/papers/rep/search.html

b http://www.genome.jp/

c http://www.ncbi.nlm.nih.gov/sites/entrez
